# Xpert MTB/RIF Assay Shows Faster Clearance of Mycobacterium tuberculosis DNA with Higher Levels of Rifapentine Exposure

**DOI:** 10.1128/JCM.01313-16

**Published:** 2016-11-23

**Authors:** A. Jayakumar, R. M. Savic, C. K. Everett, D. Benator, D. Alland, C. M. Heilig, M. Weiner, S. O. Friedrich, N. A. Martinson, A. Kerrigan, C. Zamudio, S. V. Goldberg, W. C. Whitworth, J. L. Davis, P. Nahid

**Affiliations:** a University of California, San Francisco, Division of Pulmonary and Critical Care Medicine, San Francisco, California, USA; b University of California, San Francisco, School of Pharmacy, San Francisco, California, USA; c Washington DC VA Medical Center and the George Washington University, Washington, DC, USA; d Rutgers-New Jersey Medical School, Newark, New Jersey, USA; e Division of Tuberculosis Elimination, Centers for Disease Control and Prevention, Atlanta, Georgia, USA; f Veterans Administration Medical Center and University of Texas Health Science Center, San Antonio, Texas, USA; g Division of Medical Physiology, MRC Centre for Tuberculosis Research, DST/NRF Centre of Excellence for Biomedical Tuberculosis Research, Faculty of Medicine and Health Sciences, Stellenbosch University, Tygerberg, South Africa; h Perinatal HIV Research Unit (PHRU), MRC Soweto Matlosana Collaborating Centre for HIV/AIDS and TB, University of the Witwatersrand, Johannesburg, South Africa; i Vanderbilt University School of Medicine, Nashville, Tennessee, USA; j Universidad Peruana Cayetano Heredia, Lima, Peru; k Yale School of Public Health, New Haven, Connecticut, USA; Carter BloodCare & Baylor University Medical Center

## Abstract

The Xpert MTB/RIF assay is both sensitive and specific as a diagnostic test. Xpert also reports quantitative output in cycle threshold (*C_T_*) values, which may provide a dynamic measure of sputum bacillary burden when used longitudinally. We evaluated the relationship between Xpert *C_T_* trajectory and drug exposure during tuberculosis (TB) treatment to assess the potential utility of Xpert *C_T_* for treatment monitoring. We obtained serial sputum samples from patients with smear-positive pulmonary TB who were consecutively enrolled at 10 international clinical trial sites participating in study 29X, a CDC-sponsored Tuberculosis Trials Consortium study evaluating the tolerability, safety, and antimicrobial activity of rifapentine at daily doses of up to 20 mg/kg of body weight. Xpert was performed at weeks 0, 2, 4, 6, 8, and 12. Longitudinal *C_T_* data were modeled using a nonlinear mixed effects model in relation to rifapentine exposure (area under the concentration-time curve [AUC]). The rate of change of *C_T_* was higher in subjects receiving rifapentine than in subjects receiving standard-dose rifampin. Moreover, rifapentine exposure, but not assigned dose, was significantly associated with rate of change in *C_T_* (*P* = 0.02). The estimated increase in *C_T_* slope for every additional 100 μg · h/ml of rifapentine drug exposure (as measured by AUC) was 0.11 *C_T_*/week (95% confidence interval [CI], 0.05 to 0.17). Increasing rifapentine exposure is associated with a higher rate of change of Xpert *C_T_*, indicating faster clearance of Mycobacterium tuberculosis DNA. These data suggest that the quantitative outputs of the Xpert MTB/RIF assay may be useful as a dynamic measure of TB treatment response.

## INTRODUCTION

The best available intermediate markers of tuberculosis (TB) treatment response for individual patient monitoring and for TB drug development are currently sputum smear microscopy and sputum culture conversion. Sputum culture at intermediate time points, although demonstrated in prior studies to have some utility for predicting treatment success or failure (1), has been called into question as a surrogate marker, given its limited ability to predict relapse (2, 3). Sputum smear microscopy at 2 months, though currently recommended by the World Health Organization (WHO), has a sensitivity as low as 24% for relapse (4).

The Xpert MTB/RIF assay (Xpert; Cepheid, Sunnyvale, CA), an automated, cartridge-based semiquantitative PCR (sqPCR) assay targeting the *rpoB* locus of Mycobacterium tuberculosis DNA, received WHO endorsement as a preferred TB diagnostic in 2010 (5, 6). Since endorsement, a massive rollout of Xpert has been undertaken, delivering the assay to 116 countries, with over 10 million cartridges procured under concessional pricing in the public sector (5). According to WHO estimates, the costs of Xpert are similar to those of mycobacterial culture, but large-scale implementation of Xpert will not require the infrastructure of specialized laboratories and personnel training needed for mycobacterial culture technologies (7). Some national TB programs have already replaced sputum smear microscopy with Xpert as the primary diagnostic (8–11). It is thus important to determine if there is a role for serial Xpert assays in monitoring TB treatment response. Previous studies have demonstrated that the quantitative output of Xpert in cycle threshold (*C_T_*) values, indicating the number of rounds of PCR amplification required to detect M. tuberculosis DNA, correlates closely with bacillary burden in sputum *in vitro* (12). While the routine Xpert results report includes only a semiquantitative output (negative, very low, low, medium, high), the numerical *C_T_* also may be obtained from the GeneXpert platform by an algorithm without additional software. A recent study evaluating the longitudinal use of Xpert in clinical trial participants on TB therapy found high sensitivity but very low specificity for the assay; however, this study interpreted Xpert results as a dichotomous marker (i.e., M. tuberculosis DNA detected versus not detected) compared to binary microbiologic measures of TB treatment response (i.e., smear and culture results) (13). Another study found an association between numerical *C_T_* and same-day culture status and with treatment failure (14).

In this study, we evaluated serial Xpert sputum assays over 12 weeks of TB treatment within a cohort of patients enrolled in Tuberculosis Trials Consortium (TBTC) study 29X, a phase 2 randomized clinical trial comparing dose-escalating rifapentine-based regimens with standard rifampin-based therapy for drug-sensitive TB (15). We took advantage of the parent study's pharmacokinetic (PK) measures to identify clinical and treatment-related factors associated with Xpert *C_T_* trajectory.

## MATERIALS AND METHODS

### Study sites, population, and treatments.

Of the 20 international sites participating in TBTC study 29X (ClinicalTrials registration number NCT00694629), the Xpert substudy consecutively enrolled consenting participants at 10 sites in 5 countries: Barcelona, Spain; Lima, Peru; Kisumu, Kenya; Soweto and Cape Town, South Africa; San Francisco, California, and 4 sites in Texas, United States. Adult (age, ≥18 years) ambulatory patients with smear-positive pulmonary TB participating in study 29X were enrolled. All participants underwent HIV testing. Participants were randomly assigned to one of four treatment arms, containing rifapentine at 10, 15, or 20 mg/kg of body weight or rifampin at 10 mg/kg in addition to isoniazid, ethambutol, and pyrazinamide at standard doses during the intensive phase. After completing intensive-phase treatment, participants continued treatment with a conventional continuation-phase regimen, typically isoniazid plus rifampin for 4 additional months (15, 16). Informed consent was obtained from all participants for the parent trial, and the study was approved by institutional review boards at the Centers for Disease Control and Prevention and at each participating site. None of the Xpert data obtained for this substudy were used for clinical decision-making. Detailed clinical, radiologic, and laboratory-specific information was recorded on standardized case record forms and captured using double data entry as part of the parent trial. A standardized protocol was developed and used at all 10 participating sites involved in the longitudinal evaluation of Xpert. Information regarding the design, conduct, and results of TBTC study 29X has been published previously (15).

### Sputum sample collection and processing.

Participants provided sputum samples at time of enrollment (pretreatment) and at weeks 2, 4, 6, 8, and 12 of anti-TB therapy. Per protocol, a single specimen was obtained at all time points, except for week 8 when two separate specimens were obtained. Sputum induction was performed for patients unable to expectorate. Laboratory technicians recorded the volume (which ranged to a maximum of 10 ml) and quality of each sputum sample (salivary, mucoid, or purulent) and then performed decontamination with conventional 1% to 2% *N*-acetylcysteine and sodium hydroxide methods (final NaOH concentration, 1% to 2%) (17). Centrifugation was performed for 15 min at 3,000 × *g*, and the resulting pellet was resuspended with a phosphate buffer solution, pH 6.8, to a total volume of 2 to 2.5 ml (17). For smear microscopy, 0.1 ml of the suspension was used, and 0.2 ml and 0.5 ml were inoculated into solid (Lowenstein-Jensen) and liquid (Bactec mycobacterial growth indicator tube [MGIT] 960; Becton Dickinson, Sparks, MD, USA) culture media, respectively. The residual sputum pellet was tested with Xpert using the standardized procedures described below.

### Xpert MTB/RIF procedure.

Following sampling for other microbiological outcomes, 0.5 ml of the resuspension from the residual pellet was combined with Sample Reagent (SR; Cepheid) in a 1:3 ratio, and 2 ml of sample in SR were pipetted into an Xpert MTB/RIF test cartridge. The cartridge was loaded into the instrument, and Xpert testing was performed automatically by instrument and software according to the manufacturer's recommendations. For the five target probes within the *rpoB* sequence, the sqPCR per-probe threshold cycle was archived and converted to tabular format for analysis. The assay was validated with the use of positive controls (provided by the manufacturer to each participating laboratory), prepared by spiking with a known M. tuberculosis DNA copy number, in order to verify the correct performance of the assay for the various targets.

### Statistical analysis.

Longitudinal Xpert *C_T_* data were modeled using a nonlinear mixed effects approach and performed in NONMEM version 7.3 (Icon Development Solutions, Ellicott City, MD, USA). The likelihood ratio test, which compares −2 log likelihood between two nested models, was used to assess significance. Of the five probe *C_T_* values reported for each assay, the minimum *C_T_* value was used in our analysis as recommended by the manufacturer. A likelihood-based method (M3) was implemented to handle upper censoring for sputum samples in which no M. tuberculosis DNA was detected (18). For those few patients who had multiple assay results at a single time point other than 8 weeks, replicate data were retained in our final model to increase precision. Analyses conducted with the exclusion of these replicates did not affect results. *C_T_* trajectories were best fit by a model, whereas baseline *C_T_* and linear rate of change in *C_T_* could be estimated. A baseline model for the control (rifampin) arm was developed first, followed by the addition of dose-ranging rifapentine data. Comparative *C_T_* changes for rifapentine versus rifampin were modeled as functions of regimen (dose measured in milligrams per kilogram), dose (600 mg, 900 mg, or 1,200 mg of rifapentine), or drug exposure as measured by area under the plasma concentration-time curve (AUC), which was estimated from a population pharmacokinetic model incorporating plasma rifapentine levels measured at a single time point (15).

## RESULTS

A total of 786 sputum samples were obtained from 115 consecutively enrolled study participants from weeks 0 to 12 after initiation of TB treatment. Of these specimens, 217 contained no detectable M. tuberculosis DNA and were subject to upper censoring. The bulk of undetectable specimens occurred toward the end of the study period; 30% of week 8 samples and 40% of week 12 samples were Xpert negative. Table 1 describes the demographic and clinical characteristics of the study participants.

**TABLE 1:** Demographic and clinical characteristics of Xpert study participants at time of enrollment (*n* = 115)

Characteristic	No. of participants	% of participants
Male	77	67
Age		
18–35	54	47
36–50	29	25
>50	32	28
HIV infected	8	7
CD4 lymphocyte count (cells/mm^3^)		
<50	1	1
50–199	0	0
200–350	1	1
>350	6	5
History of smoking	59	51
Body mass index (kg/m^2^)		
<16	3	3
16–18.5	37	32
18.6–25	60	52
≥25	11	10
Not reported	4	3
Race		
Asian	3	3
Black	75	65
White	25	22
Multiracial	2	2
Not reported	10	9
Cavitation on chest radiograph at enrollment	85	74
Chest radiograph class		
No cavities	30	26
Cavities, <4 cm in aggregate	40	35
Cavities, ≥4 cm in aggregate	45	39
Treatment arm		
Rifampin (10 mg/kg/day)	27	23
Rifapentine (10 mg/kg/day)	34	30
Rifapentine (15 mg/kg/day)	25	22
Rifapentine (20 mg/kg/day)	29	25
Culture negative at week 8		
Solid medium	89	77
Liquid medium	79	69
Both solid and liquid media	78	68

### Clinical factors affecting *C_T_* trajectory.

In univariate analysis, smoking within the past year and disease extent on chest radiograph were the only clinical covariates that had a significant association with Xpert results. Participants reporting any history of smoking had a lower baseline *C_T_*, indicating a higher bacterial burden at the time of enrollment (*P* < 0.01), than those who did not smoke. There were no subsequent differences in rate of change in *C_T_* based on smoking status. Subjects with a baseline chest radiograph indicating high disease extent (defined as more than half of the chest affected by TB) had a lower baseline *C_T_* and a lower *C_T_* slope on treatment, indicating higher bacterial burden at baseline (*P* = 0.05) and a slower subsequent rate of change (*P* = 0.04); however, in multivariate analyses, associations between disease extent on chest radiograph and rate of *C_T_* change were no longer significant.

### Treatment arm, drug dose, and drug exposure effects on *C_T_* trajectory.

**FIG 1:**
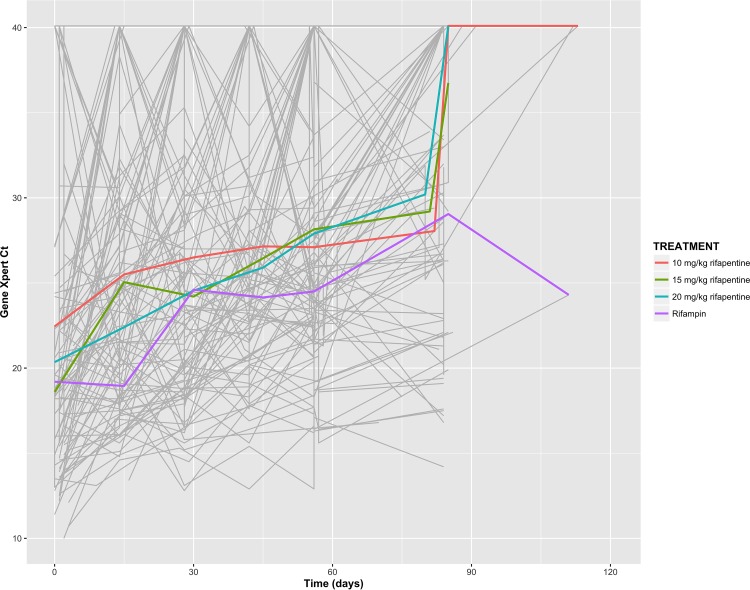
Cycle threshold (*C_T_*) trajectories for 115 individual study participants (gray lines) across treatment arms, demonstrating large intrasubject and intersubject variability over time. The estimated mean *C_T_* for each treatment arm (colored lines) rises over time, reflecting clearance of M. tuberculosis DNA with treatment. No significant difference can be seen in overall *C_T_* trajectory between treatment arms.

Median baseline *C_T_* was estimated in our model to be 19.81 (95% confidence interval [CI],18.64 to 20.98) with 24% between-subject variability. Figure 1 depicts raw *C_T_* results across treatment arms, highlighting these significant within-subject and between-subject variabilities. From week 0 to week 12, the median rate of change in *C_T_* for the rifampin arm was estimated to be 0.88 *C_T_*/week (95% CI, 0.61 to 1.14). Comparing participants who received rifampin-based therapy with all of those who received rifapentine-based therapy, irrespective of the dosing, a significant difference in *C_T_* trajectory was found, with faster M. tuberculosis DNA clearance in those receiving rifapentine at a median rate of change of 1.18 *C_T_*/week (*P* = 0.05). However, the between-subject variability across all treatment arms was high (coefficient of variation, 102%).

**FIG 2:**
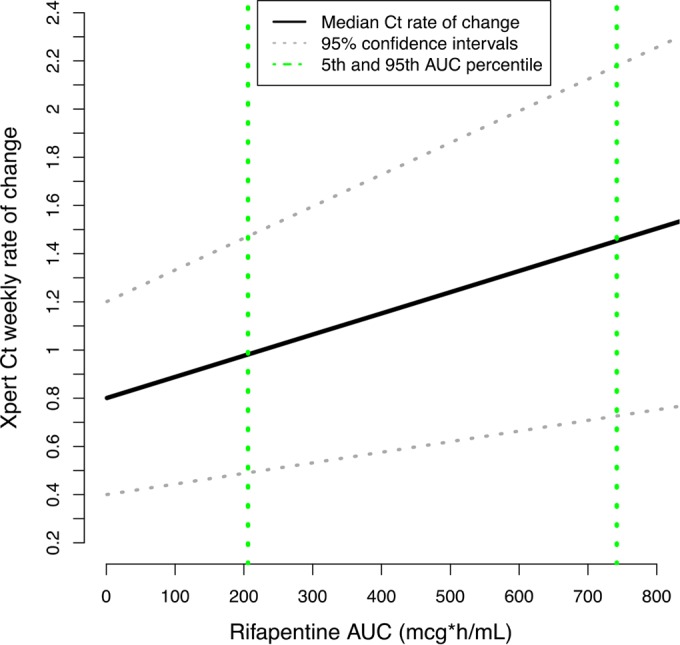
Relationship between *C_T_* slope and AUC (μg · hr/ml) in 115 study participants. As rifapentine exposure increases, rate of change in *C_T_* also increases, indicating faster M. tuberculosis DNA clearance.

Since the difference in *C_T_* trajectory between those who received rifampin and those who received rifapentine may have been due to the higher dosages of rifapentine administered during the study, we investigated the direct association of drug exposure with M. tuberculosis DNA clearance. Using a population pharmacokinetic model developed for the parent trial, rifapentine exposure (AUC) was derived for each individual (15). We found that the derived AUC was a significant predictor of the rate of change in *C_T_* (*P* = 0.02). The estimated increase in *C_T_* slope for every additional 100 μg · h/ml of rifapentine AUC was 0.11 *C_T_*/week (95% CI, 0.05 to 0.17). Figure 2 depicts the positive correlation between *C_T_* slope and median rifapentine AUC. However, *C_T_* trajectories did not vary significantly across rifapentine treatment arms (1.34, 1.38, and 1.10 *C_T_*/week for rifapentine at 10 mg/kg, 15 mg/kg, and 20 mg/kg, respectively; *P* = 0.25) or across administered dosage groups (1.19, 1.09, and 1.10 *C_T_*/week at 600, 900, or 1,200 mg, respectively; *P* = 0.13). Study 29X showed a similar association between treatment and sputum culture conversion in the liquid culture medium, in which higher rifapentine exposure, but not treatment arm (weight-based rifapentine dose) or flat rifapentine dose, was significantly associated with faster culture conversion (15). Table 2 shows previously published data by Dorman et al. (15) from their larger parent study data set cohort alongside our results from our subset of 115 study participants to demonstrate the consistent significance of rifamycin exposure across monitoring methodologies.

**TABLE 2:** Overall *P* values for comparisons of Xpert *C_T_* slope obtained in 115 substudy participants as compared to the time to stable culture conversion on a solid medium and time to stable culture conversion on a liquid medium in 195 participants in the parent clinical trial by treatment arm (rifapentine 10, 15, and 20 mg/kg versus rifampin; rifapentine dose (600 mg, 900 mg, 1,200 mg versus rifampin); and rifapentine exposure (area under the concentration time-curve tertiles)

Variable	Xpert *C_T_* slope^*a*^ (*n* = 115)	Time to stable culture conversion on solid medium^*b*^ (*n* = 195)	Time to stable culture conversion on liquid medium^*b*^ (*n* = 195)
Treatment arm	0.25	0.01	0.32
Rifapentine dose	0.13	0.01	0.38
Rifapentine exposure	0.02	<0.001	<0.001

a *P* values were derived using the likelihood ratio test based on the current substudy of 115 patients.

b *P* values were reported previously by Dorman et al. in Supplemental Table E7 (in reference 15) based on parent study cohort.

### Effects of baseline *C_T_* on subsequent rate of change.

When upper-censored data were excluded, a negative correlation coefficient of 0.57 (relative standard error, 15%) was found between baseline *C_T_* and rate of change in *C_T_* over 12 weeks, indicating faster clearance of M. tuberculosis DNA in individuals with higher baseline M. tuberculosis DNA burdens. However, this effect was no longer seen when censored data were included.

## DISCUSSION

In this study, we have shown that increasing rifapentine exposure is associated with faster M. tuberculosis DNA clearance, as measured by longitudinal Xpert *C_T_* data, over the first 12 weeks of therapy. The association found between rifapentine exposure and longitudinal Xpert *C_T_* trajectory is consistent with the biologically plausible idea that higher serum levels of effective medication lead to faster bacterial killing and supports the ability of the Xpert MTB/RIF assay to measure such a relationship. In addition, our study demonstrates the feasibility of using PK parameters as a novel alternative to comparing the assay's predictive performance with intermediate microbiologic outcomes, which can be unreliable and insensitive for predicting relapse. Interestingly, the results in this substudy mirror the data from the parent study on the relationship between drug exposure and culture conversion, suggesting that despite Xpert's lower specificity for viable mycobacteria, the overall behavior of the assay is similar to that of sputum culture in longitudinal use (15). We also found that clinical factors, including smoking status and radiographic extent of disease were associated with Xpert *C_T_* measurements in a univariate analysis. While these factors were not retained in our final statistical model based on predefined criteria, this nonetheless suggests that greater mycobacterial burden associated with these important clinical features is additionally quantified by the Xpert assay. Finally, we found that Xpert *C_T_* measurements demonstrate a high degree of within-subject and between-subject variability. Variation in Xpert's semiquantitative estimates among sputum samples within the same acid-fast bacilli grade has been previously described (19). Such variability is inherent in any quantitative sputum-based assay in which specimen collection depends on the strength of the cough, method of induction or expectoration, and many other clinical factors. Yet, even given this variability, the association between M. tuberculosis DNA clearance and rifapentine exposure was significant.

We found that a lower *C_T_* (corresponding to higher M. tuberculosis DNA load in sputum) at baseline may predict faster clearance over the course of early treatment. The Xpert MTB/RIF technology incorporates a novel filtering mechanism that primarily allows intact bacilli to be assayed in the sample, yet concerns have remained regarding the measurement of DNA from nonviable M. tuberculosis if Xpert is used in patients while on TB treatment. Our results suggest that the assay may in fact be able to measure the rapid killing of mycobacteria seen early in treatment. However, this association no longer holds true when upper-censored data, i.e., samples in which M. tuberculosis DNA is undetectable, are included. It has been shown that a minimum of 100 to 150 bacteria are required in the Xpert cartridge to classify a sample as positive for M. tuberculosis (12). This effect may therefore be subject to bias introduced by the inclusion of only observable data, and speaks to the importance of statistical methods that can incorporate nonobservable data, when dealing with data sets that have a high proportion of values outside the limits of quantification.

Our findings do not answer the question of how Xpert can be used to monitor the individual patient and predict the risk of poor treatment outcome, particularly treatment relapse. It is clear that Xpert MTB/RIF, when interpreted as a dichotomous test toward the end of the intensive phase of TB treatment, is insufficiently specific for identifying patients at high risk for poor long-term outcomes. Our findings mirror results previously published by Friedrich et al., in which Xpert remained positive in approximately 80% and 60% of patients at 8 and 12 weeks (13). However, it remains possible that Xpert cycle thresholds near or at the end of treatment at weeks 20 and beyond, when TB cultures are negative, may be informative for determining the risk of relapse.

Our study has limitations. The study contained a small proportion of HIV-infected participants, limiting its generalizability to high-HIV-prevalence populations. Second, and importantly, the parent study lacked long-term follow-up after treatment completion, and this precluded us from evaluating whether Xpert monitoring during treatment could predict a durable cure. Conversely, this study benefits from being nested within a clinical trial, which provided rigorously standardized and monitored study conditions, including the use of directly observed therapy. In addition, to our knowledge, this is the first published study to include pharmacokinetic parameters in the modeling of Xpert results. Finally, our study included patients from 10 international sites, comprising the most diverse population yet studied for Xpert longitudinal monitoring. In our results, study site was not associated with change in Xpert *C_T_*, suggesting that site-specific technical factors did not contribute to our findings.

In sum, this study demonstrates that rifapentine exposure is associated with rate of change in quantitative Xpert *C_T_* over the first 12 weeks of TB therapy, suggesting that *C_T_* may be a useful tool for monitoring treatment effect. This mirrors data from the parent trial, study 29X, which indicated faster sputum culture conversion based on rifapentine exposure rather than flat or weight-based dosage. Moreover, Xpert utilizes molecular technology that is quick, easily interpretable, and not prone to contamination compared with culture. Further evaluation of Xpert in a larger study with long-term follow-up after cessation of treatment, including later Xpert measurements, pharmacokinetics, and measures of clinically meaningful treatment outcomes, would be needed to determine the performance of this technology for predicting relapse.
